# Cellulose-Based Scaffolds with Prolonged Dexamethasone Release for Bone Tissue Engineering

**DOI:** 10.3390/molecules30132760

**Published:** 2025-06-26

**Authors:** Jolanta Liesienė, Odeta Baniukaitiene, Ieva Minseviciene

**Affiliations:** 1 Department of Polymer Chemistry and Technology, Kaunas University of Technology, 44249 Kaunas, Lithuania; odeta.baniukaitiene@ktu.lt; 2 Food Institute, Kaunas University of Technology, 44249 Kaunas, Lithuania; ieva.minseviciene@ktu.lt

**Keywords:** cellulose-based scaffolds, cellulose cationization, dexamethasone, prolonged release

## Abstract

The implantation of bone substitutes is frequently accompanied by inflammation. To reduce the inflammatory response and enhance cell adhesion, proliferation, and differentiation, scaffolds are often loaded with anti-inflammatory drugs. In this study, cellulose and cellulose/hydroxyapatite (1:1 by weight) scaffolds were developed. Structural analysis using SEM and micro-computed tomography revealed that the morphology of the scaffolds met the requirements for bone tissue engineering. The scaffolds were initially loaded with dexamethasone sodium phosphate; however, the drug was released very rapidly. To prolong its release, cationic groups were introduced into the cellulose macromolecules by amination with 2-chloro-N,N-diethylethylamine hydrochloride in an alkaline medium. Dexamethasone sodium phosphate was then immobilised on aminated cellulose and aminated cellulose/HAp scaffolds at concentrations of 157 mg/g and 87 mg/g, respectively. Due to ionic interactions between the cationic groups in the scaffolds and the anionic groups of the drug molecules, drug release was effectively prolonged. After 24 h, only about 6–7% of the drug had been released, with complete release occurring after 170 h. The cationic groups in the scaffold framework facilitated the adsorption and sustained release of dexamethasone sodium phosphate.

## 1. Introduction

Loss of bone tissue and bone defects may result from a variety of pathological and physiological conditions, including osteoporosis, hormonal imbalances, chronic inflammatory processes, tumors (e.g., bone cancer), traumatic injuries, and prolonged physical inactivity [1,2,3]. Nowadays, new possibilities have emerged for treating bone defects using bone tissue engineering [4]. The development of three-dimensional (3D) scaffolds for bone regeneration remains one of the major challenges in tissue engineering. Various materials have been proposed for fabricating 3D scaffolds, with significant attention focused on natural polymers. Cellulose serves as a valuable starting material for scaffold development due to its advantages over synthetic polymers, such as biocompatibility, non-toxicity, hydrophilicity, and slow resorption in the body [5,6,7]. To enhance osteoconductive properties, scaffolds are often prepared by combining cellulose with mineral components such as hydroxyapatite (HAp), β-tricalcium phosphate (β-TCP), cuttlebone, and others [8,9]. These mineral additives also enhance the mechanical properties of the scaffolds.

The implantation of bone substitutes is frequently accompanied by inflammation. Much attention has been given to enhancing scaffold functionality by loading them with drugs or other active compounds. To prevent inflammation and stimulate cell adhesion, proliferation, and differentiation, anti-inflammatory drugs are often immobilized within 3D scaffolds. These drugs include ketoprofen, ibuprofen, diclofenac sodium salt, dexamethasone sodium phosphate, as well as growth factors and other bioactive compounds [10,11,12,13,14,15,16,17,18].

Dexamethasone is a synthetic glucocorticoid that promotes the differentiation of osteogenic cells. It belongs to the group of immunosuppressants and anti-inflammatory drugs [19]. Due to these properties, it is one of the most commonly used drugs in tissue regeneration. Additionally, it has analgesic, anti-allergic, and immunosuppressive effects. Dexamethasone is used to treat autoimmune and rheumatological diseases and, in combination with antibiotics, for severe infectious diseases, certain types of cancer, and inflammatory eye conditions. It is also commonly used as an immunosuppressant after organ or stem cell transplantation. High doses of dexamethasone (e.g., 4 mg/kg/day) can prevent organ rejection and avoid bone marrow toxicity. Dexamethasone stimulates the expression of biochemical markers of bone metabolism, such as bone alkaline phosphatase activity and osteocalcin [16]. Dexamethasone sodium phosphate is also used in tissue engineering. Notably, 1.3 g of dexamethasone sodium phosphate is equivalent to 1 g of dexamethasone [19].

Due to their biodegradability within the body, biodegradable synthetic polymers such as poly(lactic acid) (PLLA), poly(lactic-co-glycolic acid) (PLGA), and their composites with other polymers are commonly used to fabricate resorbable scaffolds for bone tissue engineering using different methods, such as electrospinning, spray drying, solid-particle leaching, 3D printing and others. Dexamethasone is frequently immobilized within such scaffolds. The drug loading content and release rate typically depend on the nature of the scaffold used and the method of its preparation. Achieving a controlled and sustained drug release rate in physiological media remains one of the major challenges. The aim is to ensure an even release of the drug over approximately 4–7 days.

Vacanti et al. [14] immobilized dexamethasone onto PLLA or polycaprolactone (PCL) fibers produced via the electrospinning method. The diameter of the fibers without dexamethasone was 1 µm, whereas with dexamethasone, it increased to 2 µm. It was found that dexamethasone was released from PLLA fibers within one month. In contrast, drug release from PCL fibers occurred much faster, with nearly 50% released within the first 20 min and complete release achieved within 90 min. In vitro studies with mesenchymal stem cells confirmed that cell adhesion and proliferation occurred within the fibrous scaffolds. Similarly, Lee et al. [15] produced fiber-like scaffolds with immobilized dexamethasone using the electrospinning method only from PLGA. In vitro studies with adipose-derived stem cells confirmed the cytotolerance of PLGA and PLGA fibers containing dexamethasone. It was observed that the release of dexamethasone from PLGA fibers promoted the osteogenic differentiation of adipose-derived stem cells. Moreover, the influence of dexamethasone concentration on cell differentiation was evaluated, revealing that at a concentration of 10%, cell differentiation occurred faster than at lower concentrations. The researchers found that dexamethasone was completely released from PLGA fibers within 14 days.

Tang et al. [16] used spray drying to form PLLA-PEG dexamethasone microcapsules (approximately 1.22 µm in diameter) and immobilized them onto a porous PLGA scaffold. Their findings indicated that the PLGA scaffold with PLLA-PEG microcapsules was able to absorb a higher amount of dexamethasone (3 mg/g) compared to the PLGA scaffold alone (1.2 mg/g). The use of microcapsules was shown to prolong drug release from the scaffolds.

Kim et al. [17] formulated a PLGA scaffold containing dexamethasone using the solid-particle leaching method. When studying drug release kinetics, it was found that dexamethasone was released from the scaffold over four weeks, with the maximum amount released within the first four days. Additionally, in vitro studies with mesenchymal stem cells demonstrated that dexamethasone promotes cell attachment and proliferation. Yoon et al. [20] developed a biodegradable PLGA scaffold for dexamethasone immobilization using a combined gas foaming and solid-particle leaching method. The kinetics of dexamethasone release from the PLGA scaffold revealed that the drug was completely released within 30 days. Furthermore, in vitro studies with smooth muscle cells showed that the scaffold with immobilized dexamethasone supported cell proliferation. Majrashi et al. [21] successfully achieved the sustained release of dexamethasone from 3D-printed PCL scaffolds by adding the excipient sucrose acetate isobutyrate. Dexamethasone was released over 35 days in the 17–163 nM range.

Li et al. [22] prepared PLLA/PEG/nHAp composite scaffolds with dexamethasone using 3D printing and evaluated them both in vitro and in vivo. In vitro experiments with mouse MC3T3-E1 cells indicated that drug release improved late alkaline phosphatase secretion and mineralization, although it did not enhance early cell proliferation. Dexamethasone release also suppressed lipopolysaccharide-induced interleukin-6 and inducible nitric oxide synthase secretion by M1 macrophages. In vivo experiments on rat calvarial defects indicated that scaffolds containing dexamethasone promoted osteoinduction and osteogenic response.

There are very few studies on the immobilization of dexamethasone into cellulose-based scaffolds. Tsiapla et al. [23] loaded dexamethasone into cellulose acetate scaffolds fabricated through electrospinning. The release of dexamethasone from the scaffolds exhibited a biphasic pattern, with an initial burst on the first day, releasing 11.6% of the drug. The second stage was much slower and extended up to six months. According to the authors, drug release occurred through the melting of cellulose acetate fibers. Sakar et al. [24] synthesized carboxymethylcellulose/HAp nanocomposites with dexamethasone as a local drug delivery system for bone tissue engineering. The process involved several steps, starting with the preparation of in situ dexamethasone-encapsulated metal–organic frameworks, which were ultrasonically dispersed in water. This dispersion was then added dropwise to a carboxymethylcellulose/HAp slurry. The resulting three-dimensional nanocomposite was obtained by drying the dispersion in an oven at 55 °C. It was found that dexamethasone was released very slowly, sustaining release for four weeks.

In all of the aforementioned works, dexamethasone was inserted or encapsulated in polymer matrices without any bound to the matrix and its release depended on the rate of diffusion from the matrix. In order to obtain prolonged dexamethasone release, various additives or methods have been used. This work describes the immobilization of dexamethasone into three-dimensional cellulose/HAp scaffolds. Cellulose, due to its high content of OH groups, offers new possibilities for retaining dexamethasone in its matrix. By introducing appropriate functional groups into cellulose, dexamethasone can be absorbed by ionic interaction. The aim of this work is to introduce cationic groups into the cellulose and develop cellulose/HAp scaffolds with a prolonged and sustained dexamethasone release profile.

## 2. Results and Discussion

### 2.1. Cellulose and Cellulose/HAp Scaffolds and Their Structure

The scaffolds were produced by lyophilization of porous cellulose hydrogels, the preparation and properties of which are described in detail in reference [25]. The hydrogels were obtained using a novel method involving the saponification of cellulose esters and the promotion of conditions favorable for the formation of new inter- and intramolecular bonds. These hydrogels were found to possess excellent mechanical properties, with a Young’s modulus reaching up to 43 MPa. Structurally, they belong to the cellulose II modification and exhibit a semi-crystalline nature, with a crystallinity index ranging from 43% to 45%. Inverse size exclusion chromatography revealed that the hydrogels have high porosity, with pores accessible to macromolecules as large as 4 × 10^6^ Da. To preserve this high porosity, aerogels were prepared following the methodology outlined in Section 2.3. According to this procedure, the hydrogels were first immersed in a 20% ethyl alcohol solution and subsequently freeze-dried. Suitability of the obtained aerogels for bone tissue engineering has been previously confirmed by in vitro and in vivo tests [26].

It is well known that the morphology of a scaffold significantly influences the efficiency of bone tissue regeneration [27,28,29,30,31]. Studies have shown that a minimum pore size of 100 µm is required for successful bone regeneration [32]. Pores smaller than this can lead to the overgrowth of soft tissue, leaving insufficient space for hard tissue formation. Furthermore, bone regeneration cannot occur without a functional vascular network, as the circulatory system is essential for delivering nutrients to cells and removing metabolic waste. Mineral components necessary for bone formation are also transported via the bloodstream. Therefore, the formation of new blood vessels (angiogenesis) within the scaffold is crucial. Given the importance of angiogenesis, most researchers recommend that scaffold pores be larger than 350 µm but not exceed 1000 µm, with an ideal pore size of up to 700 µm [28,32]. Excessively large pores are undesirable, as they reduce the scaffold’s surface area, leading to lower cell adhesion and diminished mechanical properties [29]. Additionally, the pores must be interconnected to ensure adequate diffusion of nutrients and waste products, as well as to support vascular network formation [30]. Only 3D scaffolds made from metal alloys may be an exception, as they exhibit excellent mechanical properties even with pores larger than 1000 µm [31]. It is important to note that despite their mechanical strength, metal alloy scaffolds are hydrophobic, have poor biocompatibility, often cause inflammation in surrounding tissues, and may trigger immune rejection.

**Figure 1:**
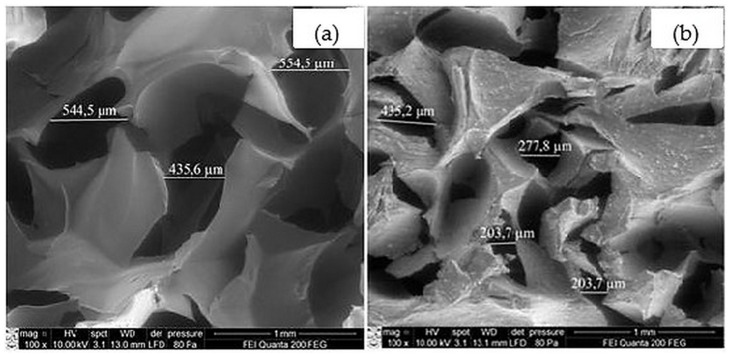
SEM images: (**a**) cellulose scaffold; (**b**) cellulose/HAp scaffold (×100 magnification).

In this study, cellulose composites with HAp, the main inorganic component of natural bone, were fabricated. The inorganic phase comprised 50% of the composite by weight. To create porous scaffolds with morphology suitable for bone regeneration, the lyophilization method was used. Scanning electron microscopy (SEM) images of the fabricated scaffolds are shown in Figure 1.

The micrographs in Figure 1 show that both the cellulose scaffold (control sample) (Figure 1a) and the composite containing HAp (Figure 1b) are porous. However, the pore size in the HAp-containing scaffold is smaller. In both the cellulose and cellulose/HAp scaffolds, the pores are asymmetric but evenly distributed.

**Figure 2:**
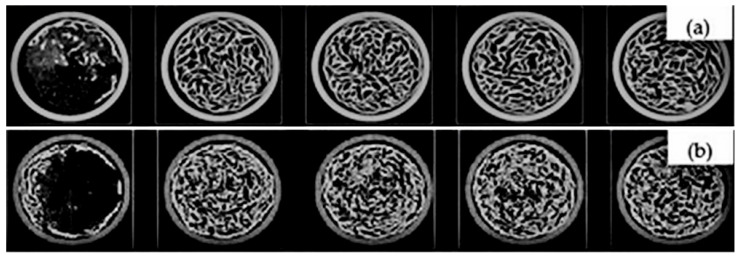
Two-dimensional slices of microCT images of cellulose (**a**); cellulose/HAp scaffolds (**b**). Scaffolds scanned in xy plane.

**Figure 3:**
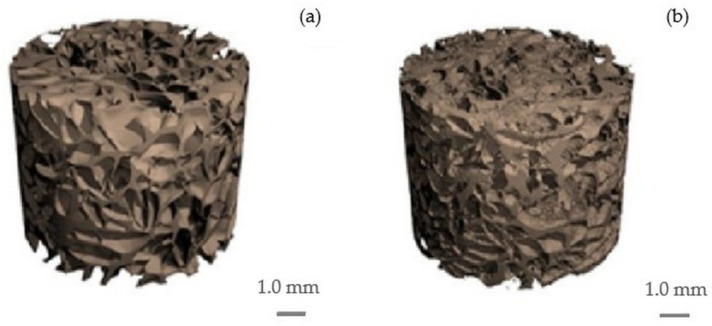
Three-dimensional microCT images of cellulose (**a**); cellulose/HAp scaffolds (**b**).

Figure 2 and Figure 3 present representative 2D and 3D images of the cellulose and cellulose/HAp scaffolds obtained via micro-computed tomography (microCT), highlighting their porous structures. The 2D images (Figure 2) clearly demonstrate that the scaffold morphology changes upon the addition of HAp particles. Not only are the pore sizes affected, but also the thickness and arrangement of the polymeric framework.

Three-dimensional microCT images of the scaffolds are shown in Figure 3. Structural parameters were identified through 3D analysis, revealing an interconnected porous architecture. The calculated parameters include the framework volume fraction (Xv), porosity (P), specific surface area (SS), mean fiber thickness (L), and mean pore diameter (D) across the entire scaffold volume. These results are summarized in Table 1.

**Figure 4:**
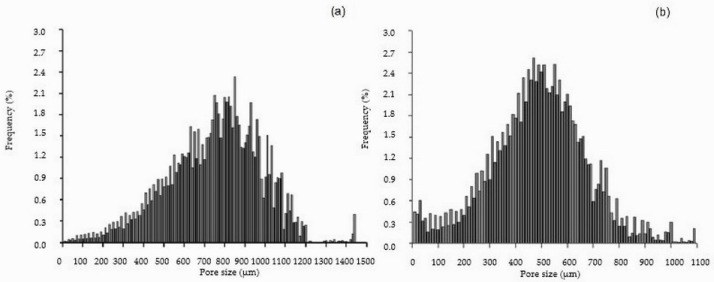
Pore size distribution in cellulose (**a**) and cellulose/HAp (**b**) scaffolds.

The data in Table 1 indicate that the pure cellulose scaffold, with a lower framework volume fraction, has higher porosity compared to the cellulose/HAp composite. The cellulose scaffold also features the largest pores (750 ± 68 µm), which explains its lower specific surface area relative to the composite. The pore size distribution histogram for the cellulose scaffold (Figure 4a) shows the presence of very large pores (up to 1440 µm), contributing to this high average. In contrast, the cellulose/HAp composite scaffold consists of thinner polymeric fibers with a higher density per millimeter and smaller pores (mean diameter: 490 µm). This structural configuration yields a higher specific surface area despite the lower overall porosity (Table 1).

The histogram in Figure 4b shows that only a small fraction of pores in the cellulose/HAp composite exceed 1000 µm in diameter—reaching up to 1090 µm and representing just 1% of the total. Compared to the cellulose scaffold, the cellulose/HAp composite has a higher proportion of pores below 300 µm (approximately 16% vs. 3%). Pore size distribution (Table 2) analysis revealed that in the cellulose/HAp composite, pores ranging from 300 to 700 µm—considered optimal for supporting tissue regeneration—constitute approximately 73% of the total, whereas in the pure cellulose scaffold, this range accounts for only 35%. Notably, pores in the 700–1000 µm range comprise 50% of all pores in the cellulose scaffold, compared to just 10% in the composite.

Overall, the morphology of the prepared scaffolds satisfies the requirements for materials used in bone defect repair, as they provide space for cells to synthesize and mineralize collagen.

### 2.2. Loading of Dexamethasone Sodium Phosphate into the Scaffolds and Its Release

**Figure 5:**
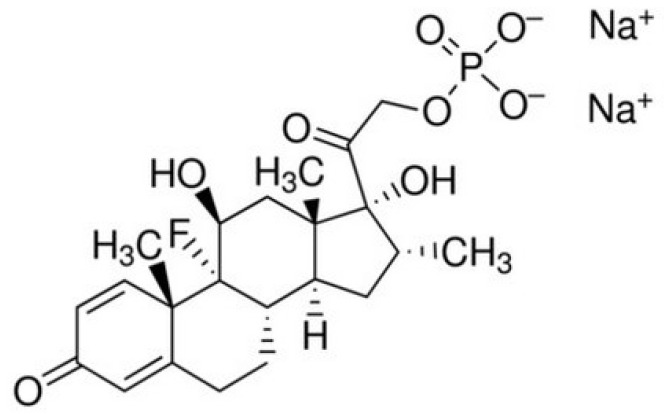
Chemical structure of dexamethasone sodium phosphate.

To improve cell adhesion, proliferation, differentiation, and reduce the inflammatory response, dexamethasone sodium phosphate (Figure 5) was loaded into the fabricated 3D scaffolds. To fill all pores, the scaffolds were kept in dexamethasone sodium phosphate solution, then frozen and lyophilised. It was found that 44 mg/g and 21 mg/g of the drug were loaded into cellulose and cellulose/HAp scaffolds, respectively.

**Figure 6:**
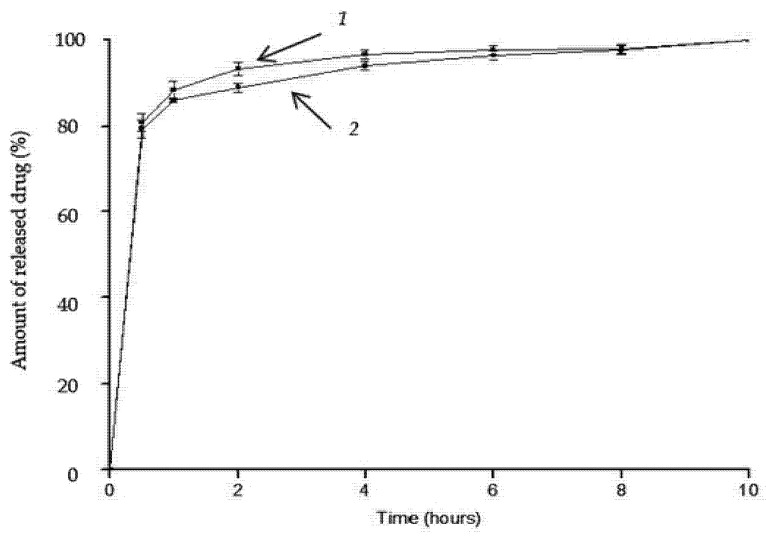
Release kinetics of dexamethasone sodium phosphate from the scaffolds: 1—cellulose; 2—cellulose/HAp composite.

The in vitro release kinetics of dexamethasone sodium phosphate from the cellulose and cellulose/HAp scaffolds show an initial burst release of the anti-inflammatory drug, which then slows down over time (Figure 6). Within just 0.5 h, approximately 81% of the drug is released, reaching 100% release after 10 h.

According to literature data [19], for optimal therapeutic effect, the anti-inflammatory drug should be released from the scaffold within four days when implanted at the site of a bone defect. Therefore, the produced composites are not suitable for extended drug release applications. This rapid release rate suggests that the drug molecules are in free form or adsorbed onto the scaffold surface by weak physical bonds.

### 2.3. Amination of the Scaffolds and Their Properties

**Figure 7:**
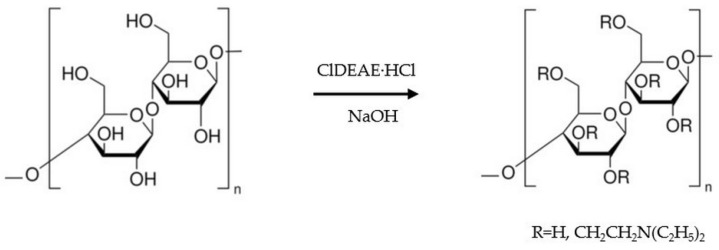
Scheme of cellulose amination.

To prolong the release of dexamethasone sodium phosphate from the scaffolds, cationic groups were introduced into the cellulose macromolecules via an amination reaction, as shown in the scheme (Figure 7). Amination was performed on cellulose gel and cellulose composites with HAp in the wet state; afterward, the gels were lyophilized.

**Figure 8:**
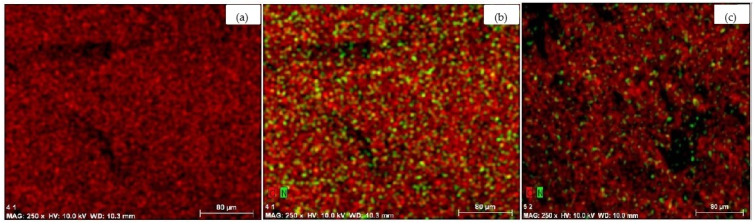
SEM-EDS images of the scaffolds: cellulose (**a**); aminated cellulose (**b**); aminated cellulose/HAp (**c**); (x250 magnification). Green colour indicates nitrogen.

The results of elemental analysis indicate that the nitrogen content in the aminated cellulose and cellulose/HAp scaffold frameworks reaches 0.72% and 0.5%, respectively. The lower nitrogen content in the composite is attributed to the reduced cellulose fraction in the sample. SEM–EDS mapping (Figure 8) confirms the presence and distribution of nitrogen throughout the scaffold. The SEM–EDS image of aminated cellulose (Figure 8b) reveals the highest nitrogen content, whereas a reduced amount is observed in the aminated cellulose/HAp composite scaffold (Figure 8c). These images validate that the amination occurred throughout the entire scaffold volume.

Mechanical stability is one of the most important factors limiting the practical application of any scaffold as a bone substitute. The compressive strength of a scaffold reflects its ability to withstand various loads without breaking down, which is especially important for bones. After testing the scaffolds, the Young’s modulus was found to be 5 MPa and 8 MPa for the aminated cellulose and aminated cellulose/HAp scaffolds, respectively. The data indicate that the cellulose scaffold without HAp exhibits lower compression resistance. The cellulose scaffold is strengthened by the immobilization of HAp particles within it. Compared to scaffolds made from synthetic polymers such as PLLA, PLGA, PCL, or their composites, the cellulose-based scaffolds are weaker. This is due to the very high porosity of the cellulose-based scaffolds and their thin polymeric framework. Interestingly, the cellulose hydrogel—the initial material for the scaffolds—is much stronger than the resulting scaffolds. The Young’s modulus of the hydrogels was found to be 41–43 MPa [25]. Considering the mechanical characteristics of the cellulose-based scaffolds, they are suitable for repairing bones that are not heavily loaded, for example, the bones of the skull.

### 2.4. Immobilization of Dexamethasone Sodium Phosphate into the Aminated Scaffolds and Its Release

Dexamethasone sodium phosphate was immobilised into aminated cellulose and aminated cellulose/HAp scaffolds at 157 mg/g and 87 mg/g of drug, respectively. Converted to the volume of the frame, this corresponds to 25.5 mg/cm^3^ and 19.8 mg/cm^3,^ respectively. A smaller amount of loaded drug in the aminated cellulose/HAp composite is influenced by a lower concentration of cationic groups in it.

**Figure 9:**
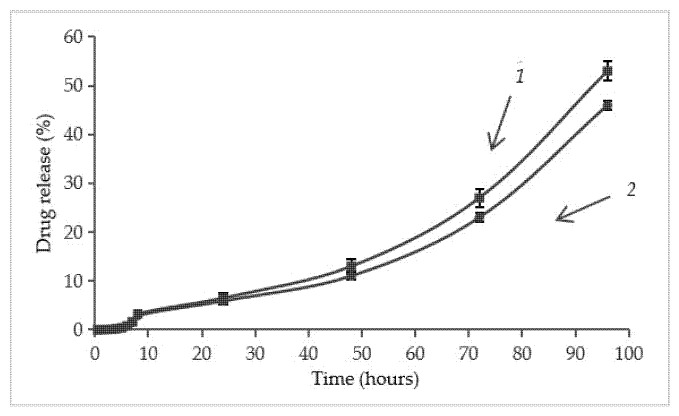
Dexamethasone sodium phosphate release from: *1*—aminated cellulose scaffold, *2*—aminated cellulose/HAp scaffold.

The release kinetics of dexamethasone sodium phosphate in vitro from the cellulose and cellulose/HAp scaffolds show an initial even release of the anti-inflammatory drug up to 50 h, which later accelerates (Figure 9). The results show that the release of the anti-inflammatory drug is very similar from both the aminated scaffolds (Figure 9). After 24 h, about 6–7% of the drug is released, and complete release occurs only after 170 h. In contrast, almost complete release of dexamethasone sodium phosphate from the non-aminated cellulose scaffold occurs during 0.5 h (Figure 6).

The prolonged release of dexamethasone sodium phosphate from aminated samples is due to the ionic interaction between cationic groups in the scaffolds and anionic groups in the drug molecules. In a physiological environment, due to ion exchange, the drug molecules are released from the framework.

## 3. Materials and Methods

### 3.1. Materials

Cellulose diacetate (degree of substitution: 2.4; 55% bound acetic acid) was kindly provided by DP Acetate Co., Kaunas, Lithuania. Aqueous ammonia (25%, chemically pure grade; CAS No. 1336-21-6) was purchased from Stanlab, Poland. Nano-hydroxyapatite (<200 nm particle size (BET), ≥97%, synthetic; CAS No.12167-74-7), dexamethasone sodium phosphate (≥98% purity; CAS No.2392-39-4), 2-chloro-N,N-diethylethylamine hydrochloride (99%; CAS No.869-24-9), and all other chemicals were purchased from Sigma-Aldrich Co., Schnelldorf, Germany. All chemicals were used without any further purification.

### 3.2. Preparation of Cellulose and Cellulose/HAp Gels

Cellulose gels were prepared according to the method described in [25]. Briefly, cellulose diacetate (CDA) was dissolved in acetone. Then, aqueous ammonia was gradually poured in. After thorough mixing, the polymer solution was placed in a tightly sealed container at room temperature for 10 days. During the hydrolysis reaction of acetyl groups, the polymer gradually lost its solubility, forming a three-dimensional network. The resulting gel was washed with water to a neutral pH of 7.

The gels with hydroxyapatite (HAp) additives were obtained by adding an appropriate amount of HAp into the CDA solution in acetone. Further procedures were the same as those for preparing cellulose gels without HAp.

### 3.3. Preparation of Cellulose and Cellulose/HAp Scaffolds

Cylindrical samples (16 mm in diameter, 10 mm in height) of the cellulose gels were soaked in 20% ethyl alcohol and cooled at −25 °C. The samples were then dried using a freeze-drying technique, ALPHA 2–4 LSC (Martin Christ Gefriertrocknungsanlagen GmbH, Hamburg, Germany), for 24 h.

### 3.4. Micro-Computed Tomography

The micro-computed tomography (micro-CT) analysis of cellulose and cellulose/HAp was performed using a µCT40 system (Scanco Medical AG, Brüttisellen, Switzerland). A scaffold sample in the form of a cylinder with a diameter of 10 mm and a height of 8 mm was scanned using the following parameters: 45 kVp energy, 600 ms integration time, 2× frame averaging, and a nominal resolution of 10 µm. To reduce image noise, the data were filtered using a constrained 3D Gaussian filter (σ = 0.8, support = 1). Both 2D and 3D images were reconstructed using software provided by the manufacturer. Quantitative evaluation of structural parameters of the scaffolds was conducted using Scanco 6.0 evaluation software.

### 3.5. Scanning Electron Microscopy and Electron Dispersive Spectroscopy

The morphology of cellulose and cellulose/HAp scaffolds was studied using a high-resolution scanning electron microscope Quanta 200 FEG (FEI, Eindhoven, The Netherlands), equipped with a Schottky-type electron gun. Images were captured at 100x magnification. The elemental composition of the scaffolds and the distribution of nitrogen atoms within the scaffolds were analyzed using an energy dispersive X-ray spectrometer, X-Flash 4030 (Bruker AXS Microanalysis GmbH, Hamburg, Germany), integrated into the microscope.

### 3.6. Preparation of the Scaffolds with Amino Groups

Cellulose was aminated with 2-chloro-N,N-diethylethylamine hydrochloride (ClDEAE·HCl) in an alkaline medium. Samples (wet gel) were immersed in the prepared solution at constant weight ratios: ClDEAE·HCl/cellulose = 0.25 and NaOH/ClDEAE·HCl = 0.32. A total of 0.001 g of NaBH_4_ was added. The reaction was carried out for 1 h with continuous stirring at 50 °C. After the reaction, the obtained product was washed with distilled water until neutral. The aminated samples were included in 20% ethyl alcohol, cooled at −25 °C, and lyophilized using a freeze-drying technique, ALPHA 2–4 LSC (Martin Christ Gefriertrocknungsanlagen GmbH, Hamburg, Germany), for 24 h.

The nitrogen content in aminated cellulose scaffolds and composites was determined by the titrimetric Kjeldahl method [33]. Dry aminated cellulose or cellulose/HAp composite samples (about 0.1 g, weighed with an accuracy of 0.0001 g) were burned in concentrated sulfuric acid in the presence of a catalyst (weight ratio SeO_2_:anhydrous CuSO_4_:K_2_SO_4_ = 1:2:18). Ammonia was distilled off with steam using a Parnas–Wagner apparatus into a flask containing a known volume of 0.005 M H_2_SO_4_ solution. After that, the contents of the flask were titrated with a 0.01 M NaOH solution. During titration, the indicator changed the color of the solution from purple to green.

The amount of nitrogen, expressed as a percentage by weight, was calculated using the following formula:

$$N=\frac{\left(V_{1}-V_{2}\right)\cdot 0.014}{m},\%$$

where *V*_1_—the volume of 0.005 M H_2_SO_4_, mL; *V*_2_—the volume of 0.01 M NaOH consumed for the titration, mL; *m*—the weight of the sample, g.

### 3.7. Loading the Scaffolds with Dexamethasone Sodium Phosphate

Cellulose scaffolds (approximately 0.2 g) were immersed in 10 mL of 6 mg/mL dexamethasone sodium phosphate solution and stored for 24 h. Samples loaded with anti-inflammatory drug were first frozen for 24 h at −25 °C and then freeze-dried using a freeze-drying technique, ALPHA 2–4 LSC (Martin Christ Gefriertrocknungsanlagen GmbH, Hamburg, Germany), for 24 h.

### 3.8. Evaluation of Dexamethasone Sodium Phosphate Release

**Figure 10:**
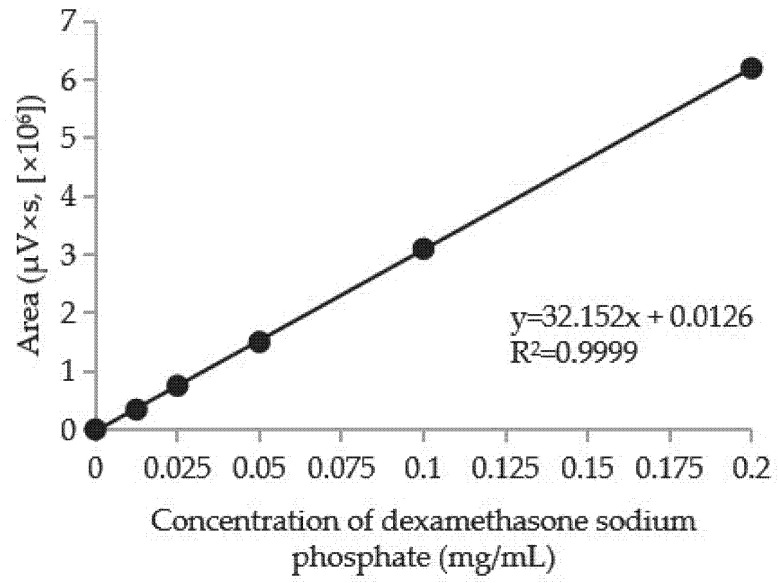
Dexamethasone sodium phosphate calibration graph.

Samples loaded with dexamethasone sodium phosphate were placed in 20 mL of 0.9% NaCl solution. At specified time intervals, 200 µL samples were collected and diluted with saline to a final volume of 20 mL. The concentration of dexamethasone sodium phosphate was determined using ultra-high-performance liquid chromatograph Shimadzu Nexera™ (Shimadzu Corporation, Kyoto, Japan). A reversed-phase chromatography column (C18, 150 mm in length and 4.6 mm in internal diameter) was used for the analysis. The sorbent particle size was 5 µm. The mobile phase consisted of a 1:1 (*v*/*v*) mixture of acetonitrile and water, with a flow rate of 1 mL/min. The injected sample volume was 10 µL. Detection was performed using a photodiode array (PDA) detector at 254 nm. In the chromatogram, the peak at 2.318 min corresponds to dexamethasone sodium phosphate [15]. A calibration graph was created (Figure 10), showing the relationship between peak area and dexamethasone sodium phosphate concentration. Based on this graph, the concentration of the anti-inflammatory drug was calculated.

### 3.9. Mechanical Properties

The compressive strength of the cellulose scaffolds was determined using a universal testing machine (H25KT, Tinius Olsen, Redhill, UK). Samples (15 mm in diameter, 5 mm in height) were subjected to a load of 5000 N at a test speed of 1 mm/min. The Young’s modulus was calculated from the stress–strain curve.

## 4. Conclusions

In this study, novel, highly porous cellulose/HAp composite scaffolds with immobilized dexamethasone were successfully developed for potential application in bone tissue engineering. To enhance dexamethasone adsorption and prolong its release, cationic groups were introduced into the cellulose macromolecules through amination with 2-chloro-N,N-diethylethylamine hydrochloride. This strategy enabled effective immobilization of dexamethasone sodium phosphate via ionic interactions, facilitating efficient drug loading within the 3D scaffold structure and allowing for controlled delivery of the anti-inflammatory agent. The aminated scaffolds exhibited a sustained drug release profile, with approximately 6% of the drug released within the first 24 h and complete release occurring after 170 h. In contrast, nearly complete release of dexamethasone sodium phosphate from the non-aminated scaffolds occurred within just 0.5 h. These findings suggest that the proposed aminated cellulose/HAp composite scaffolds hold strong potential for the localized and sustained delivery of bioactive compounds in bone repair applications.

## Figures and Tables

**Table 1:** Structural parameters of the scaffolds.

Sample	Structural Parameters				
	X_v,_ %	P, %	SS, mm^−1^	L, µm	D, µm
Cellulose scaffold	25 ± 1	75 ± 1	15 ± 2	210 ± 26	750 ± 68
Cellulose/HAp scaffold	28 ± 1	72 ± 2	19 ± 1	120 ± 10	490 ± 94

**Table 2:** Pore size distribution in the scaffolds.

Pore Size, µm	Pores, %	
	Cellulose Scaffold	Cellulose/HAp Scaffold
≥10 <300	3	16
≥300 <400	4	14
≥400 <500	7	22
≥500 <600	10	22
≥600 <700	14	15
≥700 <800	18	7
≥800 <900	18	2
≥900 <1000	14	1
≥1000 <1100	9	1
≥1100 <1500	3	0

## Data Availability

The data supporting the reported results will be made available on request.

## Abbreviations

The following abbreviations are used in this manuscript:

HAp	Hydroxyapatite
PLLA	Poly(lactic acid)
PLGA	Poly(lactic-co-glycolic acid)
ClDEAE·HCl	2-chloro-N,N-diethylethylamine hydrochloride

## References

[1] Roddy E., DeBaun M.R., Daoud-Gray A., Yang Y.P., Gardner M.J. (2018). Treatment of critical-sized bone defects: Clinical and tissue engineering perspectives. Eur. J. Orthop. Surg. Traumatol..

[2] Gage M., Liporace F., Egol K., McLaurin T. (2018). Management of Bone Defects in Orthopedic Trauma. Bull. Hosp. Jt. Dis..

[3] Tit D.M., Bungau S.G., Iovan C., Nistor-Cseppento D.C., Endres L., Sava C., Sabau A.M., Furau G., Furau C. (2018). Effects of the Hormone Replacement Therapy and of Soy Isoflavones on Bone Resorption in Postmenopause. J. Clin. Med..

[4] Porter J.R., Ruckh T.T., Popat K.C. (2009). Bone Tissue Engineering: A Review in Bone Biomimetics and Drug Delivery Strategies. Biotechnol. Prog..

[5] Budtova T. (2019). Cellulose II aerogels: A review. Cellulose.

[6] Iglesias-Mejuto A., Magariños B., Ferreira-Gonçalves T., Starbird-Pérez R., Álvarez-Lorenzo C., Reis C.P., Ardao I., García-González C.A. (2024). Vancomycin-loaded methylcellulose aerogel scaffolds for advanced bone tissue engineering. Carbohydr. Polym..

[7] Wei Z., Wu C., Li R., Yu D., Ding Q. (2021). Nanocellulose based hydrogel or aerogel scaffolds for tissue engineering. Cellulose.

[8] Palaveniene A., Songailiene K., Baniukaitiene O., Tamburaci S., Kimna C., Tihminlioğlu F., Liesiene J. (2020). The effect of biomimetic coating and cuttlebone microparticle reinforcement on the osteoconductive properties of cellulose-based scaffolds. Int. J. Biol. Macromol..

[9] Janmohammadi M., Nazemi Z., Salehi A.O.M., Seyfoori A., John J.V., Nourbakhsh M.S., Akbari M. (2022). Cellulose-based composite scaffolds for bone tissue engineering and localized drug delivery. Bioact. Mater..

[10] Kim K., Luu Y.K., Chang C., Fang D., Hsiao B.S., Chu B., Hadjiargyrou M. (2004). Incorporation and controlled release of a hydrophilic antibiotic using poly(lactide-co-glycolide)-based electrospun nanofibrous scaffolds. J. Control. Release.

[11] Chen L., Tang C.Y., Chen D.Z., Wong C.T., Tsui C.P. (2011). Fabrication and characterization of poly-D-L-lactide/nano-hydroxyapatite composite scaffolds with poly(ethylene glycol) coating and dexamethasone releasing. Compos. Sci. Technol..

[12] Canto I., Mckean R., Charnley M., Blackwood K.A., Fiorica C., Ryans A.J., MacNeil S. (2010). Development of an ibuprofen-releasing biodegradable PLA/PGA electrospun scaffold for tissue regeneration. Biotechnol. Bioeng..

[13] Tarafder S., Bose S. (2014). Polycaprolactone-coated 3D printed tricalcium phosphate scaffolds for bone tissue engineering: In vitro alendronate release behavior and local delivery effect on in vivo osteogenesis. Appl. Mater. Interfaces.

[14] Vacanti N.M., Cheng H., Hill P.S., Guerreiro J.D.T., Dang T.T., Ma M., Watson S., Hwang N.S., Langer R., Anderson D.G. (2012). Localized delivery of dexamethasone from electrospun fibers reduces the foreign body response. Biomacromolecules.

[15] Lee J.B., Jeong S.M., Kim K.J., Cho D.H., Kwon I.K., Yoon I.C., Choi K., Suh J.K.F., Park J.H., Park Y.D. (2009). Osteogenic differentiation of human adipose-derived stem cells (hADSCs) on a dexamethasone eluting nanofiber scaffolds. J. Tissue Eng. Regen. Med..

[16] Tang G.W., Yang Y.F., Sun A.P., Song T.T., Zhao Y.H., Yuan X.B., Yuan X.Y., Fan Y.B., Wang M. (2010). Controlled release of dexamethasone from porous PLGA scaffolds under cyclic loading. Sci. China Chem..

[17] Kim H., Kim H.W., Suh H. (2003). Sustained release of ascorbate-2-phosphate and dexamethasone from porous PLGA scaffolds for bone tissue engineering using mesenchymal stem cells. Biomater.

[18] Samorezov J.E., Alsberg E. (2014). Spatial regulation of controlled bioactive factor delivery for bone tissue engineering. Adv. Drug Deliv. Rev..

[19] Katzung B.G. (2003). Basic and Clinical Pharmacology.

[20] Yoon J.J., Kim J.H., Park T.G. (2003). Dexamethasone-releasing biodegradable polymer scaffolds fabricated by a gas-foaming/salt-leaching method. Biomater.

[21] Majrashi M., Kotowska A., Scurr D., Hicks J.M., Ghaemmaghami A., Yang J. (2023). Sustained release of dexamethasone from 3D-printed scaffolds modulates macrophage activation and enhances osteogenic differentiation. ASC Appl. Mater. Interfaces.

[22] Li X., Wang Y., Wang Z., Qi Y., Li L., Zhang P., Chen X., Huang Y. (2018). Composite PLA/PEG/nHA/dexamethasone scaffold prepared by 3D printing for bone regeneration. Macromol. Biosci..

[23] Tsiapla A.-R., Karagkiozaki V., Bakola V., Pappa F., Gkertsiou P., Pavlidou E., Logothetidis S. (2018). Biomimetic and biodegradable cellulose acetate scaffolds loaded with dexamethasone for bone implants. Beilstein J. Nanotechnol..

[24] Sarkar C., Chowdhuri A.R., Garai S., Chakraborty J., Sahu S.K. (2019). Three-dimensional cellulose-hydroxyapatite nanocomposite enriched with dexamethasone loaded metal-organic framework: A local drug delivery system for bone tissue engineering. Cellulose.

[25] Liesiene J., Kiselioviene S., Maruška A.S., Baniukaitiene O. Preparation and characterization of a highly porous, rigid cellulose-based hydrogel for biomedical and biotechnological applications. New J. Chem..

[26] Daugela P., Pranskunas M., Juodzbalys G., Liesiene J., Baniukaitiene O., Afonso A., Sousa Gomes P. (2018). Novel cellulose/hydroxyapatite scaffolds for bone tissue regeneration: In vitro and in vivo study. J. Tissue Eng. Regen. Med..

[27] Bose S., Vahabzadeh S., Bandyopadhyay A. (2013). Bone tissue engineering using 3D printing. Mater. Today.

[28] Wake M.C., Patrick C.W., Mikos A.G. (1994). Pore morphology effects on the fibrovascular tissue growth in porous polymer substrates. Cell Transplant..

[29] Kim K., Yeatts A., Dean D., Fisher J.P. (2010). Stereolithographic bone scaffold design parameters: Osteogenic differentiation and signal expression. Tissue Eng. Part B Rev..

[30] Karageorgiou V., Kaplan D. (2005). Porosity of 3D biomaterial scaffolds and osteogenesis. Biomaterials.

[31] Wang C., Xu D., Lin L., Li S., Hou W., He Y., Sheng L., Yi C., Zhang X., Li H. (2021). Large-pore-size Ti6Al4V scaffolds with different pore structures for vascularized bone regeneration. Mater. Sci. Eng. C.

[32] Cleynenbreugel T.V., Schrooten J., Oosterwyck H.V., Sloten J.V. (2006). Micro-CT-based screening of biomechanical and structural properties of bone tissue engineering scaffolds. Med. Biol. Eng. Comput..

[33] Anglov T., Petersen I.M., Kristiansen J. (1999). Uncertainty of nitrogen determination by Kjeldahl method. Accredit. Qual. Assur..

